# Protein acetylation derepresses Serotonin Synthesis to potentiate Pancreatic Beta-Cell Function through HDAC1-PKA-Tph1 signaling

**DOI:** 10.7150/thno.44459

**Published:** 2020-06-05

**Authors:** Yuqing Zhang, Shushu Wang, Linlin Zhang, Feiye Zhou, Kecheng Zhu, Qin Zhu, Qianqian Liu, Yun Liu, Lei Jiang, Guang Ning, Yufang Bi, Libin Zhou, Xiao Wang

**Affiliations:** 1 Shanghai Clinical Center for Endocrine and Metabolic Diseases, Shanghai Institute of Endocrine and Metabolic Diseases, Department of Endocrine and Metabolic Diseases, Ruijin Hospital, Shanghai Jiaotong University School of Medicine, Shanghai, 200025, China.; 2 School of Medicine, Cheeloo College of Medicine, Shandong University; Center for Reproductive Medicine, Cheeloo College of Medicine, Shandong University, Jinan, Shandong, 250012, China.

**Keywords:** Protein acetylation, Beta-cell function, Serotonin, Tph1, PKA, HDAC1

## Abstract

**Rationale:** Protein acetylation is tightly linked to transcriptional control and energy metabolism. However, the role of protein acetylation in islet function remains enigmatic. This study aims to determine how protein acetylation controls β-cell function and explore the underlying mechanism.

**Methods:** The gene-expression profiles were analyzed for rat islets in response to two histone deacetylase (HDAC) inhibitors. Insulin secretion, tryptophan hydroxylase 1 (Tph1) expression, and serotonin synthesis of rat islets were detected after HDAC inhibitor treatment both *in vivo* and *ex vivo.* β-cell-specific Tph1-overexpressing transgenic rats and β-cell-specific Tph1 knockout mice were constructed to evaluate the role of Tph1 in β-cell function. The deacetylation of PKA in β-cells by HDAC1 was investigated by adenoviral infection, immunoprecipitation, and western blot.

**Results:** Inhibition of HDACs greatly potentiated pancreatic β-cell function and reprogrammed transcriptional landscape of islets. Among the commonly up-regulated genes by two pan-HDAC inhibitors, *Tph1* displayed the most prominent change. Specifically, inhibition of HDAC1 and HDAC3 by MS-275 strongly promoted Tph1 expression and endogenous serotonin synthesis in rat islets, concomitantly with enhanced insulin secretory capacity *in vivo* and *ex vivo.* β-cell-specific Tph1-overexpressing transgenic rats exhibited improved glucose tolerance and amplified glucose-stimulated insulin secretion. On the contrary, β-cell-specific Tph1 knockout mice displayed glucose intolerance and impaired insulin secretion with aging. Moreover, depletion of Tph1 in β-cells abrogated MS-275-induced insulin hypersecretion. Overexpression of HDAC1, not HDAC3, inhibited Tph1 transcriptional activity and decreased MS-275-stimulated Tph1 expression. Mechanistically, HDAC1 deacetylated PKA catalytic subunit and decreased its activity, resulting in Tph1 transcriptional repression. The acetylation mimetic K62Q mutant of PKA increased its catalytic activity. HDAC1 inhibition exerted a synergistic effect with cAMP/PKA signal on Tph1 expression.

**Conclusions:** The present findings highlight a novel role of HDAC1-PKA-Tph1 signaling in governing β-cell functional compensation by derepressing serotonin synthesis.

## Introduction

The pancreatic islet β-cells serve as a glucostat to secrete insulin accordingly, especially in face of nutrient challenges 1. Protein lysine acetylation is emerging as a fine-tuned post-translational modification (PTM) that integrates metabolic flux and key physiological processes within cells 2. We previously demonstrated that lysine acetylation plays a pivotal role in modulating β-cell mitochondrial metabolism in response to glucose 3. Histone acetylation is well known for being important in chromatin regulation and gene transcription at various metabolic statuses 4. This raises the possibility that protein lysine acetylation could coordinate cell signaling network and gene expression to synchronize β-cell function to the ever-changing physiological milieu.

Reversible acetylation is driven by competing activities of histone acetyltransferases (HATs) and histone deacetylases (HDACs). The zinc-dependent HDACs have been shown to form multi-protein complexes to function as transcriptional repressors 5, among which class I and II families play critical roles in regulating metabolic gene programs 6. The past few years have implicated several HDACs in β-cell functional regulation, with negative effects of HDAC3 and HDAC7 on insulin secretion 7, 8. Meanwhile, small-molecule drugs with HDAC inhibitory activities have displayed promising potential for β-cell protection 9, 10. However, the molecular mechanism underlying protein acetylation-modulated β-cell function remains largely unknown.

Under conditions of metabolic stress such as pregnancy or high glucose, pancreatic β-cells enhance secretory capacity to compensate for the rising demand of insulin. A prominent change during this functional adaptation is the heightened synthesis and secretion of β-cell-derived serotonin (5-hydroxytryptamine, 5-HT) 11, 12, which process is mediated by the rate-limiting enzyme tryptophan hydroxylase 1 (*Tph1*). Recent studies have renewed interest in serotonin metabolism by showing that increased Tph1 expression and serotonin release augmented β-cell function and mass via 5-HT_2B_ and 5-HT_3_ receptors (HTR2B and HTR3) in insulin-resistant states 13-16. These studies established serotonin synthesis as a compensatory mechanism for β-cells in response to increased metabolic demand.

In the present study, β-cell-specific Tph1 overexpressing rats and β-cell-specific Tph1 knockout mice provided direct evidence demonstrating that Tph1-catalyzed serotonin was essential for β-cell functional compensation. To investigate how protein acetylation controls β-cell function, we analyzed the differentially expressed genes in rat islets exposed to trichostatin A (TSA) or sodium butyrate (SB), revealing a strong induction of Tph1 by the two pan-HDACs inhibitors. We further identified which HDAC was involved in the regulation of Tph1 transcription and explored the underlying mechanism.

## Results

### Inhibition of HDACs potentiates insulin secretion and reprograms transcriptional landscape in rat islets

**Figure 1:**
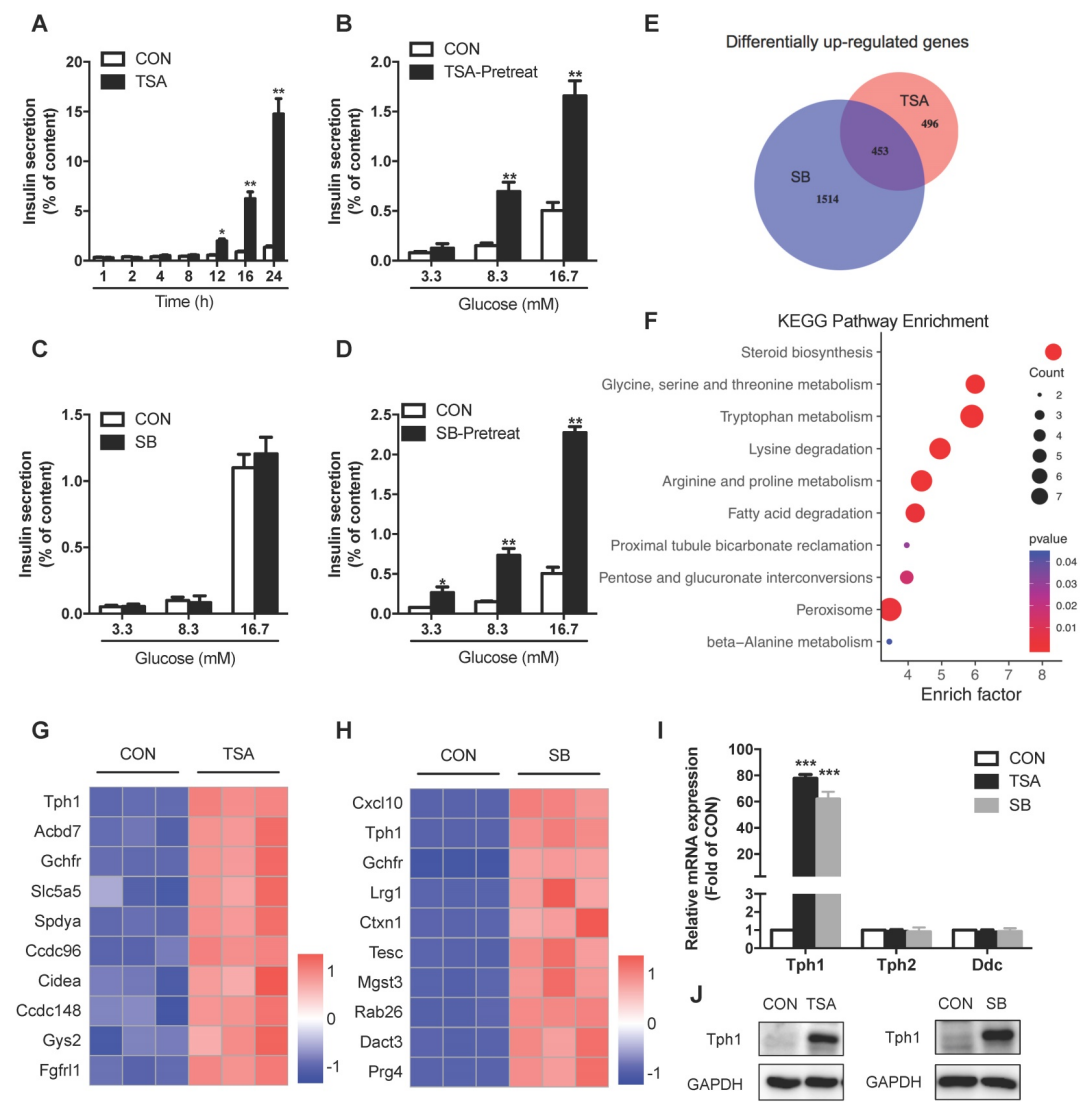
** Effects of HDAC inhibitors on insulin secretion and transcriptional landscape of rat islets. (A)** Rat islets were treated with 200 nM TSA at 3.3 mM glucose for the indicated time, and the cumulative insulin secretion was measured. **(B)** Rat islets were pretreated with 200 nM TSA at 3.3 mM glucose for 24 h, and then stimulated with 3.3, 8.3 or 16.7 mM glucose for 1 h. The supernatant was taken for insulin secretion assay. **(C)** Rat islets were stimulated with 3.3, 8.3 or 16.7 mM glucose in the presence or absence of 5 mM SB for 1 h, and insulin secretion was measured. **(D)** Rat islets were pretreated with 5 mM SB at 3.3 mM glucose for 24 h, and then stimulated with 3.3, 8.3 or 16.7 mM glucose for 1 h. The supernatant was taken for insulin secretion assay. **(E)** Comparison of the differentially up-regulated genes in TSA- and SB-treated rat islet transcriptomes. **(F)** KEGG pathway analysis of the commonly upregulated genes by both TSA and SB. **(G)** Top 10 commonly upregulated-genes in TSA-treated islet transcriptome. **(H)** Top 10 commonly upregulated-genes in SB-treated islet transcriptome. **(I)** qRT-PCR analysis of *Tph1, Tph2,* and *Ddc* mRNA expressions in rat islets incubated with 200 nM TSA and 5 mM SB at 3.3 mM glucose for 24 h. **(J)** Western blot analysis of Tph1 protein expression in rat islets incubated with 200 nM TSA and 5 mM SB at 3.3 mM glucose for 24 h. Data are expressed as mean ± SEM of three independent experiments. ^*^P<0.05, ^**^P<0.01, ^***^P<0.001 vs. control (CON).

To evaluate the effect of HDACs on islet β-cell function, we treated rat islets with TSA. Short-term TSA treatment was without effect on glucose-stimulated insulin secretion (GSIS). However, the cumulative insulin secretion was gradually enhanced with prolonged incubation of TSA, showing the most profound increase at 24 h (Figure 1A). We further pretreated rat islets with TSA for 24 h, followed by stimulation with various concentrations of glucose for 1 h. As shown in Figure 1B, TSA pretreatment induced more insulin secretion from rat islets in response to 8.3 and 16.7 mM glucose without changing insulin content (Figure S1A). To understand the molecular basis underlying the amplified insulin secretory response, the global gene-expression patterns were analyzed for rat islets incubated with or without TSA for 24 h. We detected 949 upregulated genes (fold change >2) in TSA-treated islets. Gene Ontology (GO) analysis of these genes revealed serotonin metabolism (enriched genes are *Tph1*, *Srd5a1,* and *Maoa*) as the top cellular biological process (Figure S1B). Other processes related to β-cell function including acyl-CoA metabolic process, regulation of membrane depolarization, and positive regulation of ATPase activity were also enriched. Further KEGG pathway analysis demonstrated that glycine, serine, threonine metabolism, tryptophan metabolism, and arginine, proline metabolism were significantly increased (Figure S1C).

Similar to TSA, SB inhibits the activity of class I and class II HDACs 17. SB is also a short-chain fatty acid produced by gut microbiota from dietary fiber fermentation. Butyrate has been reported to ameliorate insulin resistance due to its beneficial effect on liver and adipose tissue metabolism, whose circulating concentration is in the micromole per litre range 18, 19. In this current study, acute treatment of SB had no impact on GSIS in rat islet (Figure 1C). Pretreatment of rat islets with SB for 24 h to inhibit HDACs dramatically amplified insulin secretion in response to 3.3, 8.3, and 16.7 mM glucose without changing insulin content (Figure 1D and S1D), showing a similar action to TSA. We further performed RNA sequencing of SB-treated rat islets to examine the transcriptional profile change and identified 1967 significantly upregulated genes. The analysis of our two gene-expression profiles revealed that 453 genes were commonly upregulated by both TSA and SB (Figure 1E), whose expression may be regulated by protein acetylation in islets. These 453 differentially expressed genes were subjected to KEGG pathway analysis, showing preferentially enrichment in multiple amino acid metabolism pathways, such as glycine, serine, threonine metabolism, tryptophan metabolism, lysine degradation, and arginine, proline metabolism (Figure 1F). However, the classical genes related to glucose metabolism as well as transcription factors essential for β-cell function barely showed any change in TSA-treated islets (Figure S1E and S1F). *Tph1,* a key enzyme of serotonin synthesis, was the most profound one of TSA-upregulated genes (Figure 1G). It also ranked the second in the upregulated genes induced by SB (Figure 1H). Quantitative real time-PCR (qRT-PCR) and western blot validated a strong induction of Tph1 mRNA and protein expressions by both TSA and SB (Figure 1I and 1J). Whereas, *Tph2*, the neuronal isoform of tryptophan hydroxylase, showed no significant change. Neither did dopa decarboxylase (*Ddc*), which mediates the second step in serotonin synthesis (Figure 1I).

### HDAC1 inhibition enhances serotonin synthesis and islet β-cell function *in vivo* and *ex vivo*

**Figure 2:**
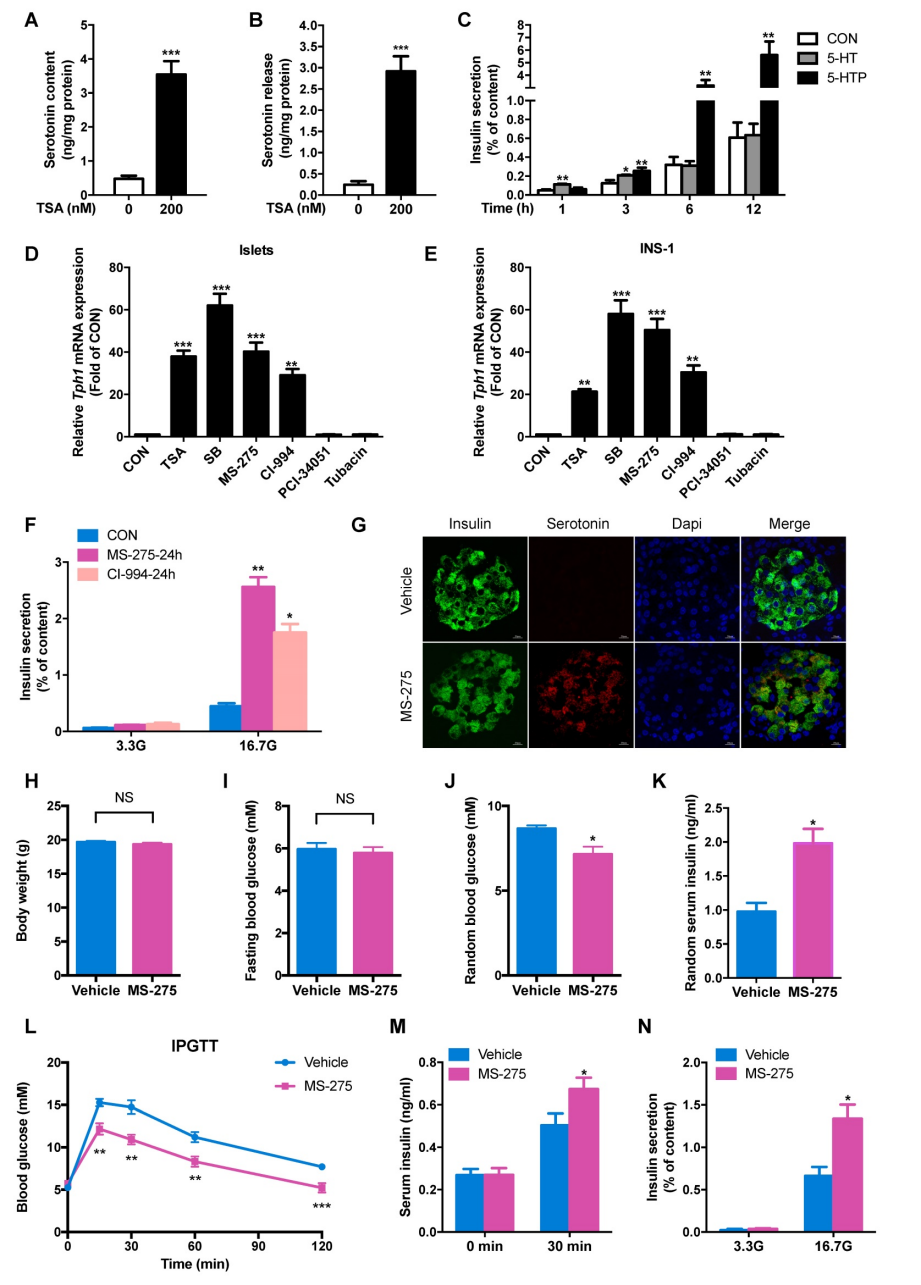
** HDAC1 inhibition increases serotonin synthesis and β-cell function of rat islets.** After rat islets were incubated with 200 nM TSA at 3.3 mM glucose for 24 h, islet serotonin content **(A)** and serotonin secretion **(B)** were measured by ELISA. **(C)** Rat islets were incubated with 500 μM 5-hydroxytryptamine (5-HT) and 500 μM 5-hydroxytryptophan (5-HTP) for the indicated time, and then insulin secretion was measured. *Tph1* mRNA expressions in rat islets** (D)** and INS-1 cells **(E)** treated with 200 nM TSA, 5 mM SB, 3 μM MS-275, 10 μM CI-994, 5 μM PCI-34051, and 10 μM Tubacin at 3.3 mM glucose for 24 h. **(F)** Rat islets were pretreated with 3 μM MS-275 and 10 μM CI-994 at 3.3 mM glucose for 24 h, then stimulated with 3.3 and 16.7 mM glucose (3.3G and 16.7G) for 1 h, and insulin secretion was measured. After mice were injected with either saline vehicle or MS-275 (20 mg/kg body weight) for consecutive 7 days, **(G)** Immunofluorescent staining was performed for serotonin (red), insulin (green) and DAPI (blue) in the pancreatic sections from mice injected with MS-275 or saline (scale bars, 20 μm). Body weight **(H)**, fasting blood glucose **(I)**, random blood glucose **(J)** and random serum insulin levels **(K)** were measured (*n*=6). **(L)** Blood glucose levels during IPGTT of mice injected with either saline or MS-275 for 5 days (*n*=6-7). **(M)** Serum insulin levels were measured before and after glucose injection from vehicle- or MS-275-treated mice (*n*=6). **(N)** Islets were isolated from mice injected with either saline or MS-275 for 7 days, then stimulated with 3.3 and 16.7 mM glucose for 1 h, and insulin secretion were measured. Data are expressed as mean ± SEM of at least three independent experiments. ^*^P<0.05, ^**^P<0.01, ^***^P<0.001 vs. control (CON or vehicle).

In parallel with increased Tph1 expression, both serotonin content and serotonin release were markedly elevated by TSA treatment in rat islets (Figure 2A and 2B). It was reported that 5-HT and its precursor, 5-hydroxytryptophan (5-HTP), exerted an opposite action on insulin secretion from insulinoma cells 20. Circulating serotonin concentration of rodent is in the range of micromole per litre 21. To definite the effects of the two metabolites on insulin secretion, rat islets were incubated with 500 μM 5-HT and 500 μM 5-HTP for various time periods. After 1 h treatment, 5-HT acutely stimulated insulin secretion, but 5-HTP was without effect. With prolonged time, 5-HT-stimulated insulin secretion was gradually attenuated while the insulinotropic action of 5-HTP appeared and enhanced (Figure 2C).

To further explore which HDAC is involved in the regulation of Tph1 transcription, we detected the effects of other HDAC-specific inhibitors. The HDAC8 inhibitor PCI-34051 and HDAC6 inhibitor Tubacin exhibited no effect on Tph1 transcription, whereas MS-275 and CI-994, two inhibitors of HDAC1, significantly increased Tph1 mRNA expression to the comparable extent as TSA and SB in rat islets and INS-1 β-cells (Figure 2D and 2E). We then treated rat islets with the two HDAC1 inhibitors to determine their action on β-cell function. MS-275 and CI-994 pretreatment for 24 h greatly potentiated GSIS without affecting cell viability (Figure 2F and S2A). Knockdown of HDAC1 in islets displayed the same effect as HDAC1 inhibitors to promote GSIS (Figure S2B and S2C). Moreover, MS-275 pretreatment significantly increased serotonin release at 3.3 and 16.7 mM glucose, especially more obvious at higher concentration of glucose (Figure S2D). As previously reported, HTR2B and HTR3 were involved in serotonin-modulated β-cell mass and function 13-16. We tested their role in HDAC1-regulated insulin secretion. MS-275-promoted GSIS was partially suppressed by the HTR3 inhibitor Ramosetron, not by the HTR2B inhibitor SB204741 (Figure S2E).

To determine whether HDAC1 inhibition improves glucose tolerance and islet β-cell function *in vivo* based on the *in vitro* findings, normal chow-fed mice were injected with either saline or MS-275 for consecutive 7 days. Serotonin staining was barely detectable in islets of control mice, whereas a marked induction for serotonin was observed in islets of MS-275-injected mice, mainly in β-cells (Figure 2G). MS-275 treatment did not affect body weight or fasting blood glucose level in mice (Figure 2H and 2I), but significantly decreased random blood glucose (Figure 2J) with a corresponding increase in serum insulin level (Figure 2K). MS-275 treatment resulted in robust improvements in glucose tolerance (Figure 2L). Meanwhile, serum insulin level 30 min after glucose injection was increased in MS-275-treated mice compared with control mice (Figure 2M). Consistent with this result, isolated islets from MS-275-treated mice released more insulin than those from control mice under the condition of high glucose (Figure 2N). These data indicate that inhibition of HDAC1 improves islet β-cell function *in vivo*.

### Enhanced glucose tolerance and insulin secretion in β-cell-specific Tph1-overexpressing transgenic rats

**Figure 3:**
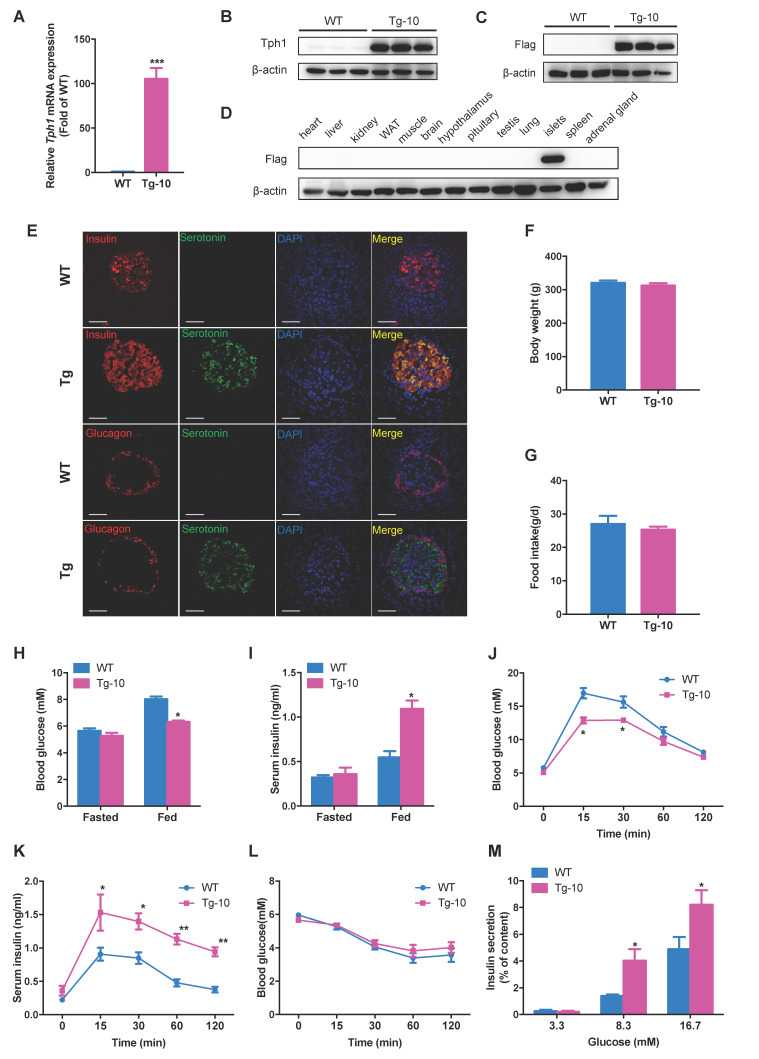
** Enhanced β-cell function in β-cell-specific Tph1-overexpressing transgenic rats. (A and B)** Tph1 mRNA (*n*=6) and protein levels in islets from wild-type (WT) and Tph1 transgenic male rat line #10 (Tg-10). **(C and D)** Flag protein level in islets and other tissues from WT and Tph1 transgenic rats. **(E)** Immunofluorescence staining for serotonin (green), insulin (red), and glucagon (red) in the pancreatic sections from WT and Tph1 transgenic rats (scale bars, 20 μm). Body weight** (F)** and food intake **(G)** of WT and Tph1 transgenic rats (*n*=10). **(H)** Fasted and fed blood glucose levels of WT and Tph1 transgenic rats (*n*=8). **(I)** Fasted and fed serum insulin levels of WT and Tph1 transgenic rats (*n*=6-7). **(J and K)** Blood glucose (*n*=8) and serum insulin (*n*=6-7) levels were measured during IPGTT from WT and Tph1 transgenic rats. **(L)** Blood glucose levels were measured at the indicated time after insulin injection (*n*=10). **(M)** Islets from WT and Tph1 transgenic rats were stimulated with various concentrations of glucose for 1 h, and then insulin secretion was assayed. All the experiments were performed on 10-week-old WT and Tph1 transgenic rats. ^*^P<0.05, ^**^P<0.01, ^***^P<0.001 vs. WT mice.

To investigate the impact of increased serotonin synthesis on β-cell function and glucose homeostasis *in vivo*, we generated transgenic rats overexpressing Tph1 under the control of rat insulin promoter (RIP). Transgenic rat line #10 (Tg-10) displayed a profound increase in Tph1 mRNA and protein expressions in islets (Figure 3A and 3B). Flag-tagged protein was detectable by Western blot in the islets of transgenic rats, not in wild-type rats or other tissues of transgenic rats (Figure 3C and 3D). Immunofluorescence staining confirmed the induction of serotonin exclusively in pancreatic β-cells of transgenic rats (Figure 3E).

The body weight and food intake in Tph1 transgenic rats were similar to those of wild-type rats (Figure 3F and 3G). No significant differences in fasting blood glucose and insulin levels were found between two groups (Figure 3H and 3I). However, fed blood glucose was markedly decreased along with raised fed insulin level in Tph1 transgenic rats (Figure 3H and 3I). We performed intraperitoneal glucose tolerance test (IPGTT) and found that glucose tolerance was significantly enhanced in Tph1 transgenic rats (Figure 3J). Meanwhile, serum insulin levels at all time points after glucose injection were increased in Tph1 transgenic rats compared with wild-type controls (Figure 3K). The insulin sensitivity was comparable between transgenic rats and their controls (Figure 3L). We further analyzed the *ex vivo* islet function of transgenic rats. The islets from Tph1 transgenic rats secreted more insulin in response to 8.3 and 16.7 mM glucose compared with those of control rats (Figure 3M). We also established another transgenic rat line #20 (Tg-20) with Tph1 overexpression (Figure S3A-C). Tg-20 rats exhibited similar phenotypes with Tg-10 rats, with improved glucose tolerance and enhanced glucose-stimulated insulin release both *in vivo* and* ex vivo* (Figure S3D-K). These findings suggest that Tph1-derived serotonin in pancreatic β-cells decreases blood glucose via potentiating the ability of β-cells to secrete insulin.

### Impaired insulin secretion with aging in β-cell-specific Tph1 knockout mice

**Figure 4:**
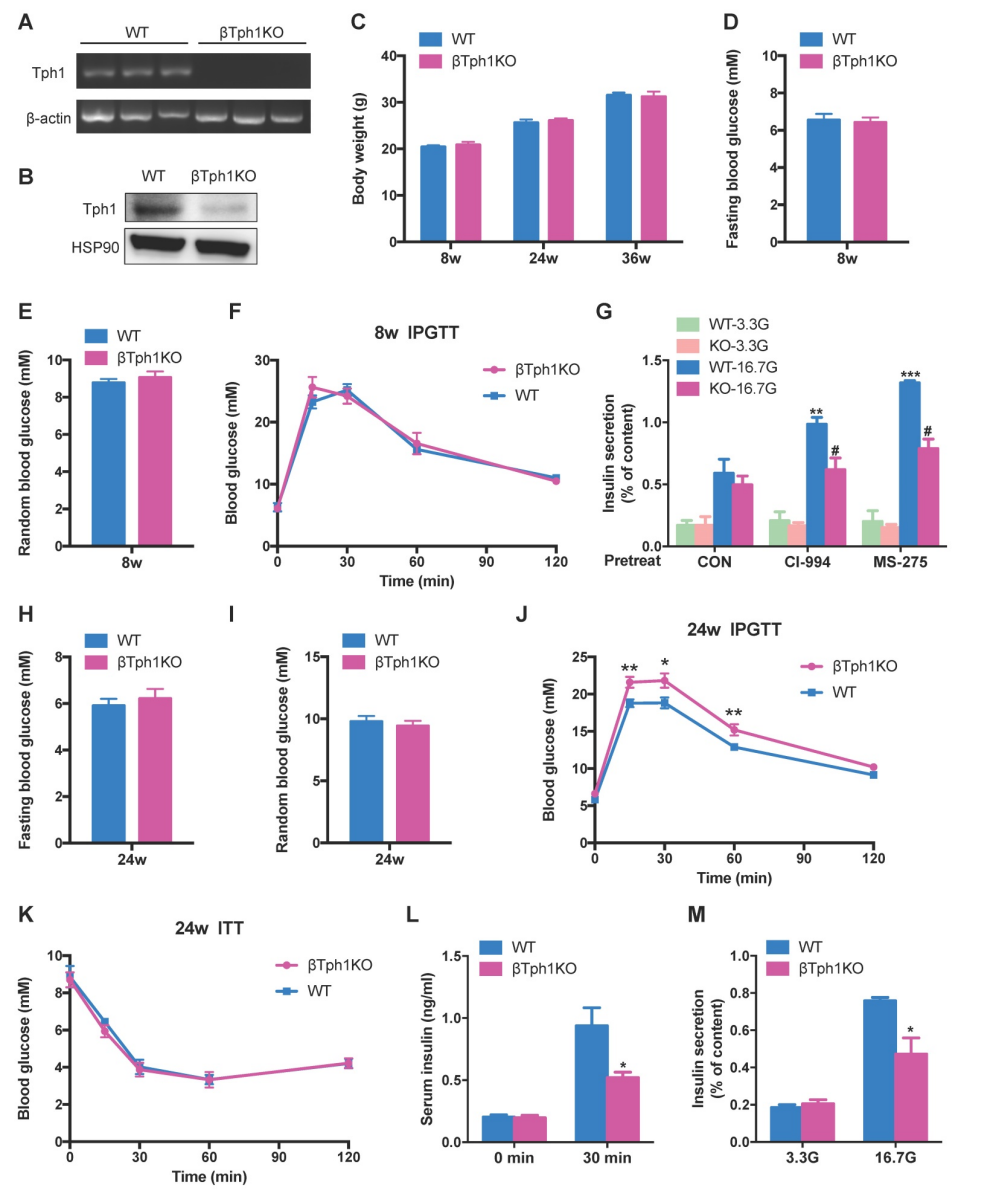
** β-cell-specific Tph1 knockout mice exhibit impaired insulin secretion. (A)** Detection of Tph1 mRNA expression in islets from WT and βTph1KO mice by RT-PCR. **(B)** Islets isolated from WT and βTph1KO mice were incubated in 16.7 mM glucose overnight, and then Tph1 protein expression was detected. **(C)** Body weight of WT and βTph1KO mice at the age of 8, 24, and 36 weeks (*n*=8-14). **(D and E)** Fasting and random blood glucose levels in 8-week-old WT and βTph1KO mice (*n*=10-12). **(F)** Blood glucose levels during IPGTT of 8-week-old WT and βTph1KO mice (*n*=6). **(G)** Islets isolated from 8-week-old WT and βTph1KO mice were pretreated with 10 μM CI-994 and 3 μM MS-275 at 3.3 mM glucose for 24 h, then stimulated with 3.3 and 16.7 mM glucose (3.3G and 16. 7G) for 1 h, and insulin secretion was assayed. **(H and I)** Fasting and random blood glucose levels in 24-week-old WT and βTph1KO mice (*n*=10-11). **(J)** Blood glucose levels during IPGTT of 24-week-old WT and βTph1KO mice (*n*=8-10). **(K)** Introperitoneal insulin tolerance tests (ITT) of 24-week-old WT and βTph1KO mice (*n*=6). **(L)** Insulin levels before and after glucose loading in 24-week-old WT and βTph1KO mice (*n*=7-9). **(M)** Islets isolated from 24-week-old WT and βTph1KO mice were stimulated with 3.3 and 16.7 mM glucose for 1 h and insulin secretion was assayed. ^*^P<0.05, ^**^P<0.01, ^***^P<0.001, vs. WT or CON. ^#^*P*<0.05 vs. WT-16.7G.

A previous study reported glucose intolerance in inducible β-cell Tph1 knockout mice under high fat diet 16. To further elucidate the role of Tph1 in β-cell functional compensation, we generated mice lacking Tph1 specifically in β-cells (βTph1KO) by crossing Tph1^flox/flox^ mice with Ins1-Cre-Dsred mice 22. Islets of βTph1KO mice displayed undetectable Tph1 mRNA and protein expressions (Figure 4A and 4B), confirming successful knockout efficiency. βTph1KO mice were born in the expected Mendelian ratio and did not exhibit a difference in body weight from their littermate controls carrying the floxed allele of Tph1 (WT) (Figure 4C). In 8-week-old mice, there were no obvious differences in fasting and random blood glucose levels (Figure 4D and 4E) between the two groups, with comparable glucose tolerance (Figure 4F). Islets from 8-week-old βTph1KO mice secreted a similar content of insulin to those from WT mice in the presence of 3.3 and 16.7 mM glucose. However, the class I HDAC inhibitors CI-994- and MS-275-potentiated GSIS was markedly reduced in βTph1KO islets compared with their WT controls (Figure 4G).

At the age of 24-week-old, although βTph1KO mice still exhibited normal fasting and fed random glucose levels (Figure 4H and 4I), they developed overt glucose intolerance compared with their controls (Figure 4J). Their random glucose level was further elevated with aging, with impaired glucose tolerance at the age of 36 weeks (Figure S4A-C). The insulin sensitivity was comparable between βTph1KO mice and their controls at both 24- and 36-week-old (Figure 4K and S4D). However, the knockout mice exhibited a poor insulin secretory response to glucose challenge (Figure 4L). Moreover, a striking reduction of GSIS was detected in islets from 24-week-old βTph1KO mice without change in insulin content (Figure 4M and S4E), suggesting that the impaired glucose regulation of βTph1KO mice is attributed to β-cell dysfunction.

### Regulation of Tph1 transcription by HDAC1 in β-cells

**Figure 5:**
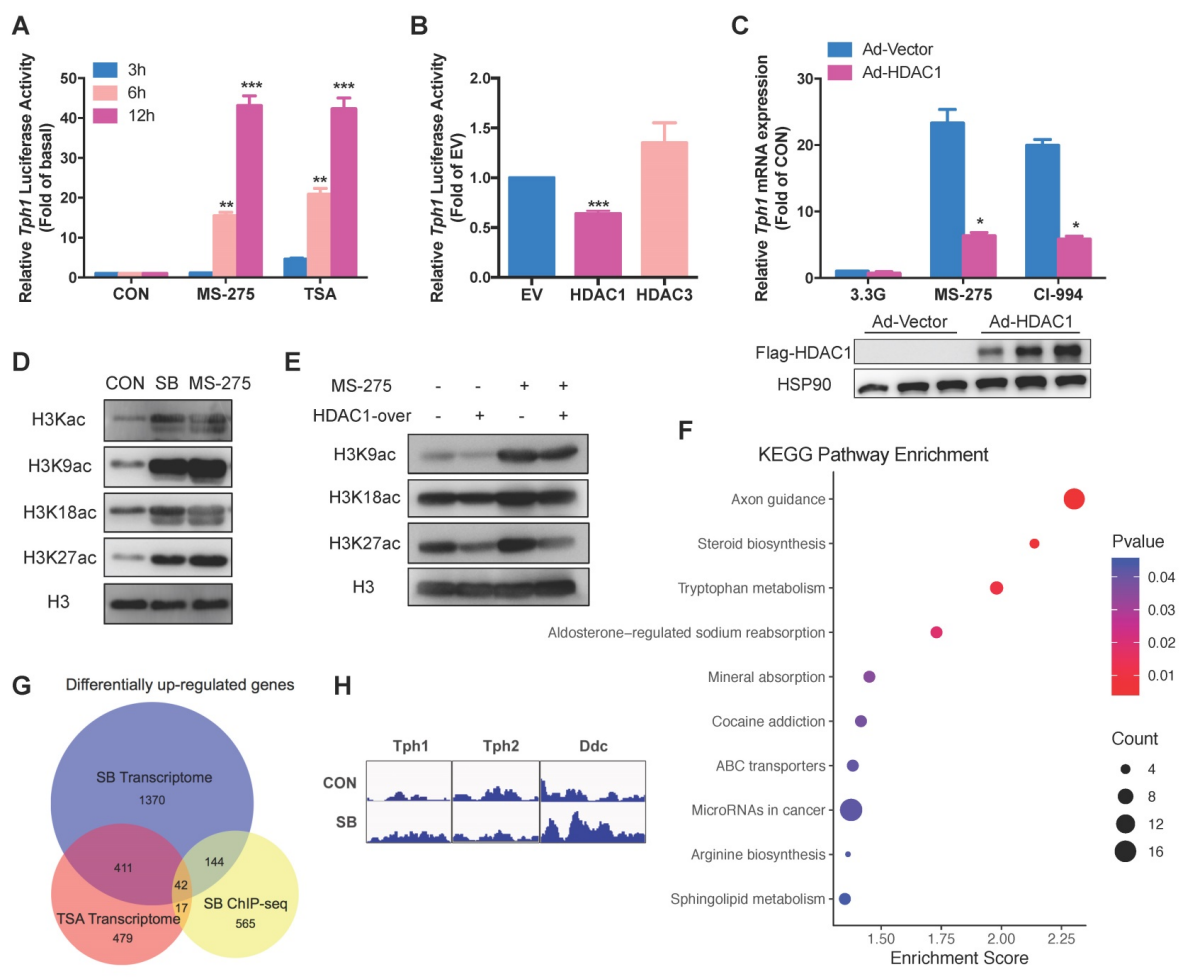
***Tph1* transcription is regulated by HDAC1 in β-cells. (A)** INS-1 cells were transfected with rat *Tph1* promoter, and then treated with 3 μM MS-275 and 200 nM TSA at 3.3 mM glucose for the indicated time. Dual-luciferase assay was performed. **(B)** *Tph1* promoter activities in INS-1 cells transfected with empty vector (EV), HDAC1 or HDAC3 plasmid. **(C)** Rat islets were transfected with vector or flag-tagged HDAC1-overexpressing adenovirus, and then treated with 3 μM MS-275 and 10 μM CI-994 for 24 h. *Tph1* mRNA expression was determined. **(D)** After INS-1 cells were treated with 5 mM SB and 3 μM MS-275 for 24 h, total H3Kac, H3K9ac, H3K18ac and H3K27ac were determined by western blot of extracted histones. **(E)** INS-1 cells were transfected with HDAC1-overexpressing adenovirus and then treated with 3 μM MS-275 for 24 h. H3K9ac, H3K18ac and H3K27ac were determined. **(F)** Top 10 upregulated KEGG pathways from ChIP-seq analysis of genes whose promoter regions were upregulated in SB-treated rat islets. **(G)** Overlap of the up-regulated genes in SB-treated islet ChIP-seq (yellow circle), TSA-treated islet transcriptome (red circle) and SB-treated islet transcriptome (blue circle).** (H)** H3K27ac patterns of *Tph1, Tph2* and *Ddc* promoters from ChIP-seq. Data are expressed as mean ± SEM of three independent experiments.^ *^P<0.05, ^**^P<0.01, ^***^P<0.001 vs. control (CON, EV, or Ad-Vector).

To observe the effect of HDAC inhibition on Tph1 transcriptional activity, a rat Tph1 promoter with luciferase was constructed and transfected into INS-1 β-cells. MS-275 and TSA increased Tph1 promoter activity with prolonged exposure (Figure 5A), consistent with Tph1 mRNA expression patterns (Figure 2E). As MS-275 exerts an inhibitory effect on both HDAC1 and HDAC3 activities 23, we next determined which one was responsible for Tph1 transcription. As illustrated in Figure 5B, only HDAC1 overexpression decreased Tph1 promoter activity in INS-1 cells. Moreover, adenovirus-mediated HDAC1 overexpression in rat islets markedly suppressed MS-275- and CI-994-induced Tph1 expression (Figure 5C), indicating that HDAC1 mediates acetylation-modulated Tph1 transcription. It is well established that HDACs control gene expression through modifying histone marks 24. We then examined whether the acetylation statuses of histone H3 at lysine 9 (H3K9), lysine 18 (H3K18), and lysine 27 (H3K27) were regulated by HDAC1 in β-cells. The HDAC inhibitors SB and MS-275 increased acetylation levels of all three sites in INS-1 cells (Figure 5D). But HDAC1 overexpression only significantly decreased the acetylation level of H3K27 induced by MS-275 (Figure 5E), suggesting that H3K27 may be a critical site for Tph1 transcription.

To address this hypothesis, we performed chromatin immunoprecipitation sequencing (ChIP-seq) analysis using antibody against H3K27 acetylation (H3K27ac) in rat islets treated with a pan-HDAC inhibitor SB. ChIP-seq identified 1981 genes whose H3K27ac were upregulated and 1304 genes downregulated by SB (fold change >2), among which 768 genes showed increased enrichment of promoter regions. KEGG pathway analysis of these genes revealed that tryptophan metabolism was significantly enriched (Figure 5F), in consistent with RNA-seq result (Figure 1F). We further compared these 768 genes with the differentially up-regulated genes in TSA- and SB-treated islet transcriptomes. 59 genes were revealed to be overlapped with TSA transcriptome while 186 genes were overlapped with SB transcriptome (Figure 5G). Of the commonly up-regulated genes by the two HDAC inhibitors, only 9 percent (42 out of 453) were found in ChIP-seq dataset. Unexpectedly, Tph1 and Tph2 promoter regions showed comparable H3K27ac level after SB treatment, whereas H3K27 in promoter regions of Ddc was hyperacetylated (Figure 5H). These findings suggest that there exists a non-histone acetylation mechanism involved in Tph1 transcriptional regulation.

### HDAC1 controls Tph1 transcription through deacetylating PKA catalytic subunit in β-cells

**Figure 6:**
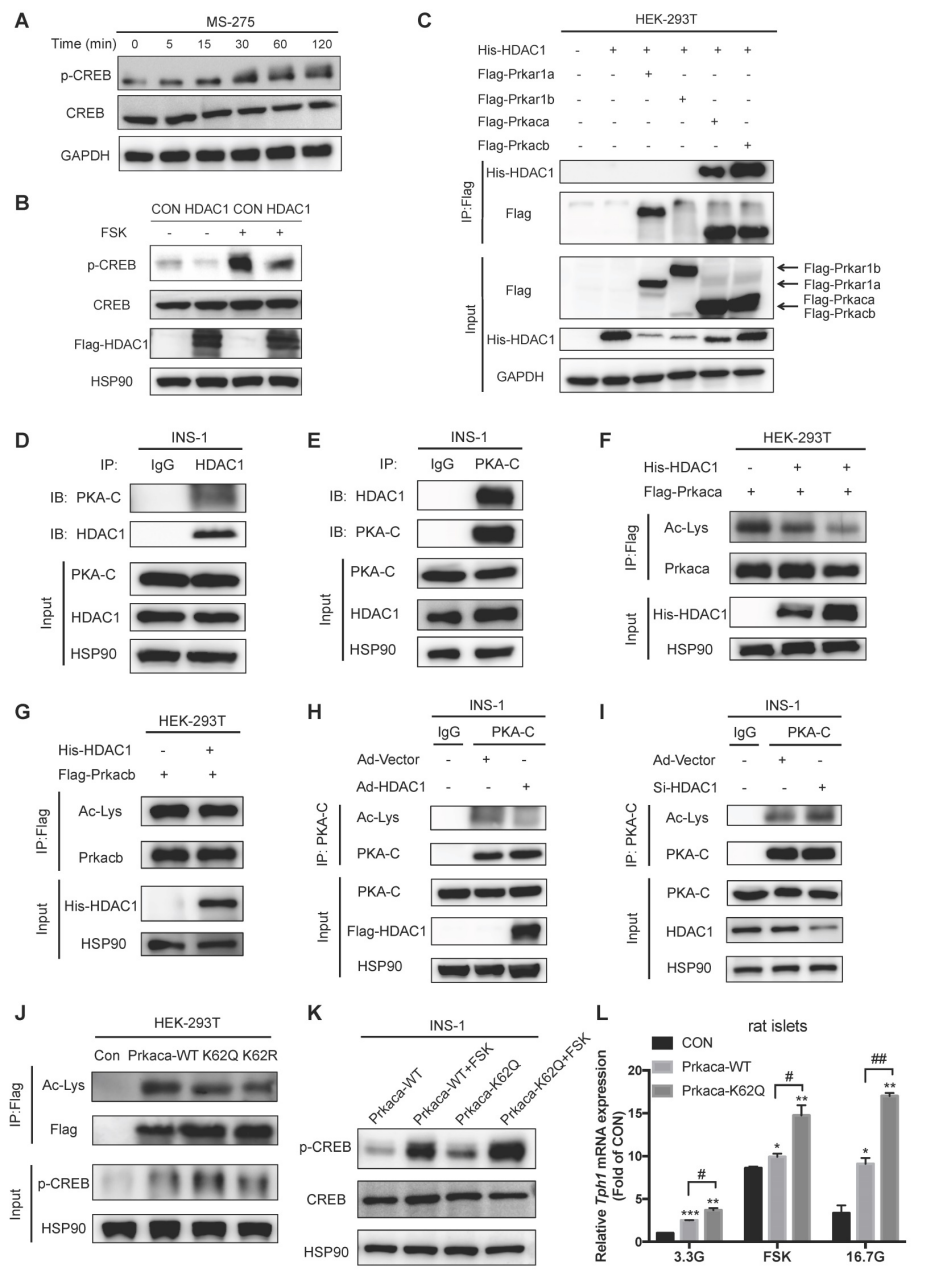
** HDAC1 deacetylates and regulates the catalytic activity of Prkaca. (A)** CREB Ser133 phosphorylation levels in INS-1 cells treated with 3 μM MS-275 for the indicated time. **(B)** INS-1 cells were transfected with control vector (CON) or HDAC1-overexpressing adenovirus (HDAC1), and then stimulated with or without 5 μM foskolin (FSK) for 1 h. CREB phosphorylation level was assayed by western blot. **(C)** His-tagged HDAC1 and flag-tagged Prkar1a, Prkar1b, Prkaca or Prkacb were co-expressed in HEK-293T cells. The co-immunoprecipitated His-tagged HDAC1 was detected in Flag beads purified proteins. **(D)** Western blot detection of endogenous PKA-catalytic subunit (PKA-C) level of immunoprecipitated HDAC1 from INS-1 cells. **(E)** Western blot detection of endogenous HDAC1 level of immunoprecipitated PKA-C from INS-1 cells. Acetylation levels of ectopically expressed Prkaca **(F)** and Prkacb **(G)** in HDAC1-overexpressing HEK-293T cells. **(H)** Acetylation level of endogenous PKA-C in INS-1 cells transfected with vector or HDAC1-overexpressing adenovirus. **(I)** Acetylation level of endogenous PKA-C in INS-1 cells transfected with vector or HDAC1 silencing adenovirus. **(J)** Flag-tagged wild-type, K62Q or K62R mutant of Prkaca was overexpressed in HEK-293T cells and the levels of Prkaca acetylation and CREB phosphorylation were determined. **(K)** INS-1 cells were transfected with wild-type or K62Q of Prkaca adenovirus and then treated with 5 μM FSK at 5.6 mM glucose for 1 h. CREB Ser133 phosphorylation was determined. **(L)** Relative *Tph1* mRNA expression levels in rat islets transfected with control vector, Prkaca wild-type (WT) or K62Q mutant adenovirus in the presence of 3.3 mM glucose, 3.3 mM glucose plus 5 μM FSK or 16.7 mM glucose for 24 h. Data are expressed as mean ± SEM of three independent experiments. ^*^P<0.05, ^**^P<0.01, ^***^P<0.001 vs. control (CON). ^#^*P*<0.05, ^##^*P*<0.01.

Our previous study demonstrated that the transcription factor cAMP-response element binding protein (CREB) activated Tph1 gene expression via binding to its promoter 12. We next sought to investigate whether HDAC1 affects cAMP/PKA/CREB signaling in β-cells. Notably, MS-275 treatment elicited CREB phosphorylation at serine 133 in a time-dependent manner (Figure 6A). HDAC1 overexpression markedly decreased the cAMP-elevating agent foskolin (FSK)-stimulated CREB phosphorylation (Figure 6B). This was the case for the phosphorylation of PKA substrate (Figure S5A) whereas cAMP level showed no significant change in β-cells in response to HDAC1 inhibition (Figure S5B). These findings strongly suggest that HDAC1 exerts a regulatory role by targeting PKA signaling in β-cells. Therefore, we screened our global acetylation proteome of rat islets and revealed several PKA subunits such as Prkar1a, Prkar1b, Prkaca, and Prkacb to be acetylated 3. To identify which of these proteins are the bona fide targets of HDAC1, we ectopically co-expressed the distinct PKA subunits and HDAC1 in HEK-293T cells. Co-immunoprecipitaion (Co-IP) experiment showed that only Prkaca and Prkacb interacted with HDAC1 (Figure 6C). Moreover, there existed an interaction of endogenous PKA catalytic subunit (PKA-C) with HDAC1 in INS-1 cells (Figure 6D and 6E). Lysine acetylation could be detected in both Prkaca and Prkacb (Figure S5C). HDAC1 co-expression only decreased the acetylation level of ectopically-expressed Prkaca, not Prkacb (Figure 6F and 6G), indicating that Prkaca is the deacetylation target of HDAC1. Similarly, HDAC1 overexpression decreased the acetylation level of endogenous PKA-C in INS-1 cells (Figure 6H). HDAC1 knockdown showed an opposite action (Figure 6I). Thus, it is reasonable to suppose that HDAC1 is the deacetylase of PKA-C.

Prkaca was identified to be acetylated at lysine 62, lysine 93, and lysine 293 (K62, K93, and K293) in our islet acetylome 3. By searching the UniProt protein database, we found that K62 was near the ATP binding region of Prkaca. To test whether the acetylation of Prkaca changes its catalytic activity, we therefore generated the glutamine and arginine mutants of Prkaca K62 (K62Q and K62R). Both K62Q and K62R mutants presented diminished acetylation signals (Figure 6J), suggesting that K62 is the major site of acetylation in Prkaca. As illustrated in Figure 6J, Prkaca overexpression stimulated CREB phosphorylation. Phosphorylation level of CREB was further increased by the acetylation mimetic K62Q, not by the non-acetylable K62R mutants. This was the case in INS-1 cells (Figure 6K). Additionally, FSK-stimulated CREB phosphorylation was enhanced in Prkaca K62Q-expressing INS-1 cells (Figure 6K). In line with this, Tph1 expression was upregulated in K62Q-expressing rat islets and INS-1 cells (Figure 6L and S5D). Moreover, either FSK- or high glucose-elicited Tph1 transcription was further augmented by acetylated K62Q of Prkaca compared with wild-type control (Figure 6L). Collectively, these results confirm that K62 acetylation increases the catalytic activity of PKA.

### Synergistic effect of HDAC1 inhibition and cAMP/PKA signal on Tph1 expression

**Figure 7:**
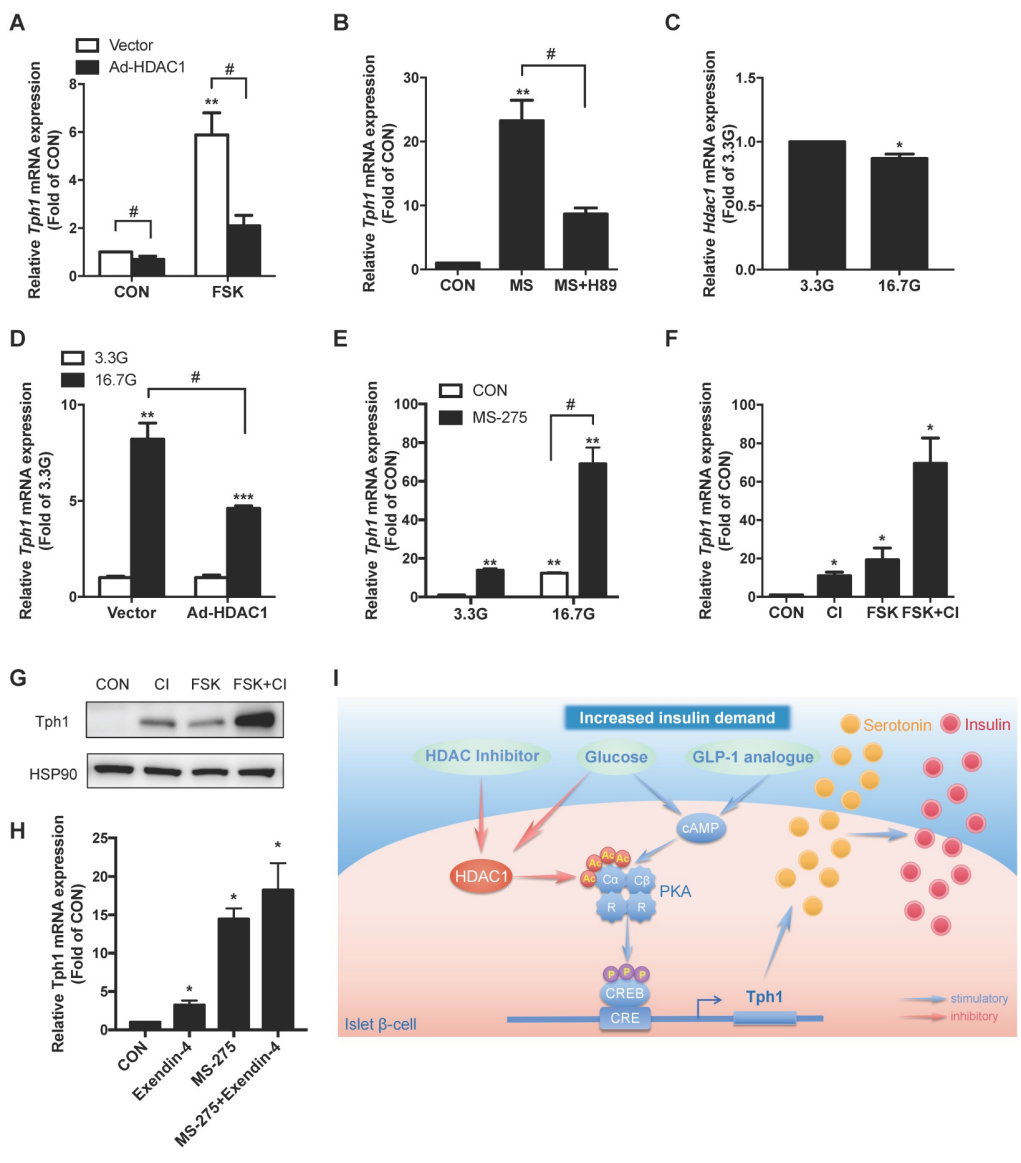
** Synergistic effect of HDAC1 inhibition and cAMP/PKA signal on Tph1 expression. (A)** Relative *Tph1* mRNA levels in rat islets transfected with control vector or HDAC1-overexpressing adenovirus in the presence or absence of 5 μM forskolin (FSK) at 5.6 mM glucose for 24 h. **(B)** *Tph1* mRNA expression in rat islets incubated with 3 μM MS-275 (MS) in the presence or absence of 10 μM H89 for 24 h. **(C)** Rat islets were treated with 3.3 and 16.7 mM glucose (3.3G and 16. 7G) for 24 h, and then *Hdac1* mRNA expression was detected. **(D)** Rat islets transfected with control vector or HDAC1-overexpressing adenovirus were further treated with 3.3 and 16.7 mM glucose for 24 h, and *Tph1* mRNA expression was detected. **(E)** *Tph1* mRNA expression in rat islets incubated with 3 μM MS-275 in the presence of 3.3 or 16.7 mM glucose for 24 h. Tph1 mRNA **(F)** and protein expressions **(G)** in rat islets incubated with 10 μM CI-994 (CI) and 5 μM FSK for 24 h. **(H)** *Tph1* mRNA expression in rat islets incubated with 10 nM Exendin-4 and 3 μM MS-275 for 24 h. **(I)** The schematic illustration summarizes the effect of protein acetylation on insulin secretion via HDAC1-PKA-Tph1 signaling. In the basal state, HDAC1 binds to and deacetylates Prkaca, thus disrupting CREB phosphorylation to repress Tph1 transcription. Under the condition of increased insulin demand, HDAC1 inhibition increases acetylation level of Prkaca, leading to CREB phosphorylation and subsequent Tph1 transcriptional derepression. This could further augment glucose- or GLP-1 analogue-elicited Tph1 expression. Tph1-mediated serotonin production triggers the adaptive insulin hypersecretion from β-cells. Data are expressed as mean ± SEM of three independent experiments. ^*^P<0.05, ^**^P<0.01 vs. control (CON) or 3.3G.^ #^*P*<0.05.

Given that HDAC1 controls PKA catalytic activity, we then assessed the contribution of the two signals to Tph1 transcriptional regulation. HDAC1 overexpression markedly suppressed FSK-stimulated Tph1 mRNA expression in rat islets (Figure 7A). Inhibition of PKA with H89 also dramatically decreased MS-275-elicited Tph1 expression (Figure 7B and S5E). Glucose is the main driver for β-cell metabolism and survival. Our previous work demonstrated that high glucose stimulated Tph1 expression through a cAMP/PKA-dependent pathway 12. We found that HDAC1 expression level of islets was significantly decreased after high glucose treatment (Figure 7C). As expected, overexpression of HDAC1 significantly suppressed glucose-elicited Tph1 expression in rat islets (Figure 7D). Moreover, HDAC1 inhibitor MS-275 exerted a synergistic effect with high glucose on Tph1 transcription (Figure 7E). CI-994 and FSK induced Tph1 mRNA expression by 11- and 19-fold, respectively. Of interest, CI-994 treatment combined with FSK stimulated Tph1 transcription up to 69-fold (Figure 7F), demonstrating a synergistic effect. Western blot showed a similar result for Tph1 protein expression in the presence of the two agents (Figure 7G). It is well known that glucagon-like peptide-1 (GLP-1) promotes pancreatic β-cell function and mass via a cAMP/PKA-dependent pathway 25. Consistently, the long-acting GLP-1 analogue exendin-4-stimulated Tph1 mRNA expression was further augmented by MS-275 (Figure 7H and S5F). Taken together, these results further support the notion that HDAC1 inhibition potentiates PKA signaling to induce serotonin synthesis in islets.

## Discussion

The present study dissected the comprehensive transcriptional landscape induced by HDAC inhibition in islets and uncovered an unexpected mechanism underlying protein acetylation-enhanced β-cell function via increasing serotonin synthesis. By using β-cell-specific Tph1-overexpressing rats and β-cell-specific Tph1 knockout mice, we confirmed that Tph1-drived local serotonin production accounted for the adaptive functional compensation of islet β-cells. Additionally, we identified HDAC1/PKA signaling as a novel regulatory axis for Tph1 expression, thus providing a potential therapy for type 2 diabetes characterized by impaired β-cell functional compensation.

Emerging findings highlight the regulatory potential of protein lysine acetylation in various biological processes by linking energy metabolism to cell signaling 26. HDACs are critical epigenetic regulators of metabolic signatures 6, 27. They are often recruited to specific genes and modify chromatin structure by deacetylating histone tails, resulting in gene repression 28, 29. All 11 classical HDACs (HDAC1-11) are expressed in the pancreatic beta cells 30. Increasing evidence reveals that HDACs regulate pancreatic β-cell function and glucose homeostasis 31-33. The deletion of HDAC3 in adult mouse β-cells improves glucose tolerance via increasing insulin secretion 8. HDAC7 overexpression resulted in impaired glucose-stimulated insulin secretion, which was restored by inhibiting HDAC7 activity 7. It was reported that class IIa HDAC4, 5, and 9 were involved in the control of the β/δ-cell development 31. However, another study displayed no alteration for insulin secretion in INS-1E cells after overexpressing wild-type HDAC4 and 5 or dominant negative HDAC4 and 5 34. Many HDAC inhibitors exhibited a protective action for pancreatic β-cells against cytokines- and streptozotocin-induced injury 10, 35, 36. In our study, two pan-HDAC inhibitors TSA and SB potentiated insulin secretion from rat islets in the absence of cytokines. Apparently, global protein hyperacetylation contributes to the enhancement of pancreatic β-cell function.

Although protein acetylation is tightly linked with islet function, its long-term impact on the regulation of gene transcription in islet β-cells remains elusive. HDAC3 depletion in beta-cells identified upregulated genes involving in amino acid activation, anion transport and calcium binding, without altering insulin gene expression 8. HDAC4 and HDAC5 have been reported to regulate β-cell development by changing expression of transcriptional factors such as *NeuroD1, Pdx1,* and *MafA* 31. HDAC7-regulated genes participate in DNA replication and repair, nucleotide metabolism, and protein folding 7. Our two gene expression patterns in rat islets treated with two pan-HDAC inhibitors identified a bulk of upregulated genes involving in multiple amino acid metabolism as well as lipid metabolism pathways. It is particularly intriguing that certain amino acids including glycine, arginine, glutamate, alanine, and tryptophan have been demonstrated to stimulate insulin secretion 37-39. However, the genes essential for classical glucose metabolism as well as insulin gene expression stayed unchanged in our transcriptome, in line with the studies on HDAC3, -4, -5 and -7 7, 8, 31. Therefore, it is reasonable to suppose that HDAC inhibition reprograms β-cell metabolism for enhanced insulin secretion by activating the metabolic fluxes of these unconventional amino acids and lipids, which may be silent at basal status. Among these TSA- and SB-upregulated genes controlling amino acid metabolism, Tph1, a rate-limiting enzyme for the biosynthesis of serotonin, displayed the most profound change, implicating its involvement in acetylation-altered metabolic signature of islets.

Recent studies reiterate the importance of peripheral serotonin by revealing its role in maintaining energy homeostasis 21, 40, 41. Human and rodent pancreatic β-cells expressed all genes required to synthesize, package, and secrete serotonin 42, 43. Placental lactogen induced Tph1 expression and serotonin production in mouse β-cells during pregnancy, resulting in increased functional beta cell mass 11, 44. In this current study, 5-HT rapidly stimulated insulin secretion, but its action only lasted a relatively short time. On the contrary, 5-HTP gradually potentiated insulin secretion with prolonged administration. As 5-HTP could be efficiently converted to serotonin in β-cells 20, 45, it is logical that it takes time for extracellular 5-HTP to enter β-cells for the continuous synthesis of serotonin, which enhances insulin secretion in an autocrine manner. Notably, serotonin is undetectable in rodent β-cells under normal physiological conditions. It is almost exclusively produced and secreted under conditions of increased metabolic demand, such as pregnancy or high glucose exposure 12, 15, 44. Moreover, its synthesis is dramatically elevated in β-cells of obese individuals with higher incidence of insulin resistance 42. Therefore, the increased serotonin signaling is required for islets to cope with higher metabolic challenge by secreting more insulin accordingly. Our study unraveled that MS-275 promoted serotonin synthesis and enhanced β-cell function via eliciting Tph1 expression in islets. Conversely, HDAC1 but not HDAC3 overexpression repressed Tph1 transcription in INS-1 cells, indicating that HDAC1 is responsible for acetylation-mediated β-cell adaptive plasticity by adjusting Tph1-catalyzed serotonin flux. HDAC1 inhibitors such as MS-275 have already been tested in many clinical trials for anti-cancer treatment 23, 46, thus they could be clinically effective tools to enhance β-cell function through triggering serotonin production, as supported by amplified insulin response in MS-275-injected mice.

Although it has been demonstrated that Tph1-catalyzed serotonin is required for beta-cell adaptive compensation in response to pregnancy or metabolic challenge, little direct evidence exclaims the role of islet-derived Tph1 in beta-cell function *in vivo*. Even more, β-cell-specific Cre mouse lines with the minigene human growth hormone (*hGH*), which strongly induced Tph1 expression and serotonin biosynthesis, displayed impaired GSIS 47. Therefore, we constructed the Tph1 transgenic rat model specifically expressed in beta-cells. These transgenic rats exhibited improved glucose tolerance as well as enhanced GSIS both *in vivo* and *ex vivo*, solidly confirming the insulinotropic action of β-cell-derived serotonin signaling. Serotonin has been proposed to act in an autocrine manner through 5-HT_3_ and 5-HT_2b_ receptors to stimulate insulin secretion 13, 14. Our study showed that 5-HT_3_ receptor partially mediated HDAC1 inhibition-potentiated insulin secretion. Alternatively, intracellular serotonin could modulate insulin secretion by protein serotonylation 20, which may also explain the increased GSIS in our Tph1 transgenic rats. The global Tph1 knockout mice exhibited hyperglycemia as early as 14 d after birth 20. But Kim et al. reported comparable glucose tolerance and GSIS in 10-week-old inducible βTph1KO mice and wild-type mice under normal chow 16. In this study, we conditionally knocked out Tph1 by using Ins1-Cre mice without *hGH* expression and observed similar phenotypes with Kim's work when these mice were young, as serotonin signaling was not necessarily required at this normal state. However, HDAC1 inhibition-induced insulin hypersecretion was greatly attenuated in these βTph1KO islets, supporting the idea that serotonin signaling is critical for acetylation-potentiated β-cell function. When βTph1KO mice reached 24 weeks, they developed overt glucose intolerance, along with defective insulin secretory response to glucose. This impaired glucose homeostasis was exacerbated in 36-week-old mice. Aging of mice is accompanied by increased peripheral insulin resistance as well as augmented insulin secretion 48, implying that beta-cell compensation is necessary to maintain euglycemia in aged mice 49. Therefore, it is possible that the serotonin signaling becomes more important for β-cells to keep up with this increased insulin demand during aging. Tph1 depletion might lead to age-related glucose intolerance because of β-cell functional decompensation.

Acetylation of histones plays important roles in balancing transcriptional output in response to metabolic changes 50. HATs catalyze the addition of acetyl group onto lysine side-chains of histones, weakening interactions between histones and DNAs and allowing access to the transcription machinery 51. Classically, HDAC1 was thought to be recruited to chromatin predominantly by transcriptional repressors to remove this histone mark and facilitate transcriptional repression 52. In our study, acetylation of H3K27 was identified to be regulated by HDAC1 in beta-cells. However, ChIP-seq analysis of this histone mark did not detect any change in Tph1 promoter after SB treatment. As only a fraction of genes regulated by HDAC inhibitors were changed in the H3K27ac ChIP-seq, it is possible that other histone acetylation marks or non-histone acetylation mechanisms are involved in HDAC1-regulated transcription. Protein acetylation has recently emerged as a widespread modification on broad classes of proteins, ranging from transcription factors to metabolic enzymes, ribosomal proteins and kinases 26, 53. Interestingly, our study revealed that Prkaca was the target of HDAC1. Like MS-275, the acetylation mimetic mutant of Prkaca stimulated CREB Ser133 phosphorylation as well as Tph1 expression. These data support the idea that HDAC1 disrupts CREB phosphorylation to repress Tph1 transcription via deacetylating Prkaca (Figure 7I). PKA resides at the center of the cAMP signaling hub that controls β-cell function, proliferation, and survival 54, 55. The anti-diabetic insulinotropic hormone GLP-1 potentiates glucose-dependent insulin release via the cAMP/PKA pathway 56. Strikingly, HDAC1 overexpression suppressed both forskolin- and high glucose-elicited Tph1 expression. Moreover, both glucose- and GLP-1 analogue exendin-4-stimulated Tph1 expressions were further augmented by MS-275, indicating that HDAC1 inhibition is a potential means to trigger Tph1-mediated β-cell functional compensation (Figure 7I).

Protein acetylation interacts actively with other PTMs in an agonistic or antagonistic manner to form codified signaling programs 57. Our findings provide a novel mode of crosstalk between acetylation and phosphorylation by effectively working in synergy to control PKA-Tph1 signaling pathways for adaptive β-cell function. As phosphorylation is usually the first wave of cell signaling event in response to stimuli, this kind of crosstalk provides an effective way to convert acetylation-based actions to phosphorylation-based signals for regulating cellular function. Apart from the calcium and cAMP signals 12, β-cell Tph1 expression was also reported to be regulated by JAK2-STAT5 pathway as well as the transcription factor MAFB during pregnancy 44, 58. Whether HDAC1-mediated acetylation interplays with other signaling pathways to regulate Tph1 expression is warranted for further evaluation.

In summary, our study demonstrates that HDAC inhibition enhances β-cell function by stimulating Tph1 transcription and serotonin synthesis. HDAC1 inhibits cAMP signal-stimulated CREB phosphorylation and Tph1 expression via deacetylating PKA catalytic subunit. Tph1-drived serotonin production in islets is required for maintaining glucose homeostasis in the process of aging via eliciting β-cell functional compensation. Thus, the modulation of HDAC1-PKA-Tph1 signaling in β-cells could serve as a novel therapeutic strategy for the treatment of type 2 diabetes.

## Methods

### Islet isolation, cell culture and treatment

Pancreatic islets were isolated from 8-12-week-old male Sprague-Dawley rats as described previously 3. INS-1 cells (passage 22-35) were cultured in RPMI 1640 medium with 11.1 mM glucose that contained 10% FBS. HEK293T cells were cultured in DMEM medium with 10% FBS. Plasmid transfection was carried out by Lipofectamine 2000 (Invitrogen). TSA was purchased from Cell Signaling. Sodium butyrate, forskolin, H89, exendin-4, 5-HT, 5-HTP, and SB204741 were obtained from Sigma-Aldrich. MS-275, CI-994, PCI-34051, Tubacin, and Ramosetron were purchased from Selleck.

### Animals

To generate β-cell-specific Tph1 transgenic rats, a flag-tagged complete coding sequence of rat Tph1 cDNA was introduced in pRP.ExSi-rat insulin I promoter and microinjected into fertilized oocytes of Sprague-Dawley rats (Cyagen Biosciences Inc. Guangzhou). Transgenic rats were identified by PCR genotyping from tail DNA using forward and reverse primers 5'-CGTCCAATGAGCGCTTTCTGC-3' and 5'-ACTCAATATGTAACAGGTTCACG-3', respectively. Six transgenic founders were established and crossed to wild-type Sprague Dawley rats (Shanghai Laboratory Animal Company, Shanghai, China). Offspring from line #10 and #20 exhibiting high copy numbers of Tph1 were used for subsequent analyses. Male rats were used in all the experiments.

To generate Tph1 floxed mice, targeting vector with the exon 3 of mouse Tph1 gene flanked by LoxP sites as well as the Neo cassette was engineered and electroporated into ES cells (Cyagen Biosciences Inc. Guangzhou). ES cells containing the floxed allele (after Neo removal) were then microinjected into fertilized oocytes from C57BL/6 mice. Genotypes of floxed mice were determined by PCR analysis of tail DNA using forward and reverse primers 5'-CTTCCCTGAGACTCAGTGCTCCTT-3' and 5'-GGAAGCAGACTGCTGAGGCTAAGG-3', respectively. β-cell-specific Tph1 knockout (βTph1KO) mice were generated by crossing Tph1^flox/flox^ mice with Ins1-Cre-Dsred mice (C57BL/6J background) as previously described 22. Male βTph1KO (Tph1^flox/flox^ Ins1-Cre) mice were used for experiments and their littermates (Tph1^flox/flox^) were used as wild-type controls. All animals were fed *ad libitum* and housed in facility on a 12 h light-dark cycle. All procedures were performed in accordance with the approval of the Animal Care Committee of Ruijin Hospital, Shanghai Jiaotong University School of Medicine.

For MS-275 treatment, 8-10-week-old wild-type C57BL/6 mice were injected intraperitoneally with MS-275 (20 mg/kg body weight daily) or vehicle (saline) for 5 days before performing glucose tolerance test. Then mice were injected for two more consecutive days before measurements of body weight, blood glucose, and insulin levels, and then islet isolation was performed.

### Metabolic studies

For intraperitoneal glucose tolerance test, mice or rats were fasted overnight, and glucose was injected intraperitoneally at 2 g/kg body weight. Blood samples were taken from a tail vein at the indicated time points for glucose and insulin measurements. Glucose levels were measured by a portable glucometer (Accu-Chek Performa, Roche). Serum insulin levels were measured using Mouse/Rat Ultrasensitive Insulin ELISA kits (ALPCO). For insulin tolerance test, mice or rats were fasted for 6 h, and insulin (Humulin R, Eli Lilly S.A.S) was injected intraperitoneally at 0.75 U/kg body weight. Blood glucose levels were determined from tail vein bleeds at 0, 30, 60, 90, and 120 min.

### Insulin secretion assay

Isolated islets were cultured with the indicated reagents in RPMI 1640 medium containing 0.25% BSA. To stimulate insulin secretion, islets were preincubated in Krebs-Ringer buffer (KRB) containing 3.3 mM glucose for 30 min. Groups of ten islets in triplicates were then incubated with KRB buffer containing either 3.3, 8.3 or 16.7 mM glucose as indicated for 1 h at 37°C. The supernatants were collected for measurement of insulin secretion. Islets were then extracted with 0.18N HCl in 70% ethanol and centrifuged to determine total insulin content. Insulin levels were measured using an ELISA kit (Mercodia, St Charles, MO) and insulin secretion was normalized as percentage of total insulin content.

### Microarray expression analysis

Total RNA was extracted from isolated rat islets incubated with or without 200 nM TSA for 24 h using Trizol (Invitrogen). Sample labeling and array hybridization were performed according to the instructions for the Agilent One‑Color Microarray‑Based Gene Expression Analysis protocol (Agilent Technologies, Inc., Santa Clara, CA, USA). Total islet RNA (1 μg) was amplified and transcribed into fluorescent cRNA using the manufacturer's Agilent's Quick Amp Labeling protocol (version 5.7, Agilent Technologies). The labeled cRNAs were hybridized onto the Whole Genome Oligo Array (4×44K, Agilent Technologies). After having washed the slides, the arrays were scanned by the Agilent Scanner G2505C. Agilent Feature Extraction software (version 11.0.1.1) was used to analyze acquired array images. Quantile normalization and subsequent data processing were performed using the GeneSpring GX v11.5.1 software package (Agilent Technologies).

### RNA sequencing analysis

Total RNA was extracted from isolated rat islets incubated with or without 5 mM SB for 24 h. Three independent biological replicates were included in the analysis. After removing cRNA, total mRNA was used to generate the cDNA library using KAPA Stranded RNA-Seq Library Prep Kit (Illumina), which was then sequenced on the Illumina HiSeq 4000. RNA-seq reads were mapped to the rat genomes. Genes with corrected *p* value less than 0.05 and fold change greater than 2 were assigned as significantly differentially expressed.

### RNA isolation and qRT-PCR

Total islet RNA was extracted using RNeasy Plus Mini kit (Qiagen, GmBH, Germany) followed by cDNA synthesis using reverse transcription kit (Promega, Madison, USA). Real time-PCR was performed with SYBR Premix Ex Taq (Takara, Kyoto, Japan) on a Light-Cycler 480 instrument (Roche Applied Science). For each gene, mRNA expression was calculated relative to that of *18S* or *β-actin*. The primer sequences were shown in Table S1.

### Western blot and immunoprecipitation

Isolated islets or cells were lysed, blotted and developed as previously described 12. Primary antibodies are listed as following: anti-Tph1 (Abcam), anti-HDAC1 (Abcam), anti-CREB (Cell Signaling), anti-phosphorylated CREB Ser133 (Cell Signaling), anti-phosphorylated-(Ser/Thr) PKA substrate (Cell Signaling), anti-PKA catalytic subunit (Abcam), anti-Flag tag (Sigma-aldrich), anti-His tag (Sigma-aldrich), anti-H3 (Cell Signaling), anti-H3K9ac (PTM Biolab), anti-H3K18ac (PTM Biolab), anti-H3K27ac (PTM Biolab), anti-GAPDH (Proteintech), anti-β-actin (Proteintech), anti-α-tubulin (Proteintech), anti-HSP90 (Cell Signaling).

Immunoprecipitation was performed by incubating protein lysates with anti-FLAG M2 Affinity Gel (Sigma-aldrich) overnight at 4°C. Endogenous immunoprecipitation was carried out by incubating lysates with the indicated antibodies for 2 h and then with Protein A/G PLUS-Agarose (Santa-Cruz) overnight at 4°C. The binding complexes were washed with lysis buffer and then eluted with loading buffer. Standard western blotting was followed using anti-acetyllysine (PTM Biolab) antibody for acetylation detection or other antibodies indicated above.

### Serotonin immunostaining and measurement

Pancreatic sections embedded in paraffin were deparaffinized, rehydrated, and incubated overnight at 4°C with rabbit anti-serotonin (1:100, Immunostar), guinea pig anti-insulin (1:400, Dako), and mouse anti-glucagon (1:200, Sigma) primary antibodies, and then detected with FITC-labeled antiguinea pig IgG (1:200, Jackson ImmunoResearch) and rhodamine-coupled anti-rabbit IgG (1:200, Jackson ImmunoResearch) secondary antibodies.

After rat islets were incubated with the HDAC inhibitors for 24 h, the medium and islet lysates were taken for serotonin assay by a commercial serotonin ELISA kit (LDN, Nordhorn, Germany) and normalized by the total protein concentration.

### Cell viability and cAMP assay

For cell viability assay, 10 μl of CCK-8 (Beyotime) was added into each well for 2 h and plates were processed in accordance with the instructions. Cellular cAMP assay was performed using an ELISA kit (Abcam) following the manufacturer's instruction.

### Histone extraction

INS-1 cells were washed with phosphate-buffered saline (PBS) and collected using the EpiQuik Total Histone Extraction Kit (EpiGentek, NY, USA) according to the manufacturer's instructions. Briefly, cells were suspended in Pre-lysis buffer at 10^7^ cells/ml and lysed on ice for 10 min. After centrifuging at 3000 rpm for 5 min at 4°C, the supernatant was discarded. Then cells were re-suspended in three volumes of lysis buffer and incubated on ice for 30 min. After centrifuging at 12000 rpm for 5 min, the supernatant was moved to a new tube and 0.3 volumes of DTT and balanced salt solution at a ratio of 1: 500 were added to the supernatant. After extraction, histones were denatured and stored at -20°C for use.

### ChIP-sequencing

INS-1 cells treated with or without 5 mM SB for 24 h were fixed with 1% formaldehyde and lysed. Three biological replicates of ChIP were performed using H3K27ac antibody and protein A agarose. Cross links were reversed and digested, followed by DNA isolation. 10 ng of DNA samples were prepared for Illumina sequencing as the following steps: 1) DNA samples were blunt-ended; 2) A dA base was added to the 3' end of each strand; 3) Illumina's genomic adapters were ligated to the DNA fragments; 4) PCR amplification was performed to enrich ligated fragments; 5) Size selection of ~200-1500bp enriched product using AMPure XP beads. The completed libraries were quantified by Agilent 2100 Bioanalyzer. The libraries were denatured with 0.1 M NaOH to generate single-stranded DNA molecules, captured on Illumina flow cell, amplified *in situ*. The libraries were then sequenced on the Illumina HiSeq 4000. After the sequencing platform generated the sequencing images, the stages of image analysis and base calling were performed using Off-Line Basecaller software (OLB V1.8). After passing quality filter, the clean reads were aligned to Rat genome (UCSC RN5) using BOWTIE software (V2.1.0). Aligned reads were used for peak calling of the ChIP regions using MACS V1.4.2. Statistically significant ChIP-enriched regions (peaks) were identified by comparison of IP vs Input or comparison to a Poisson background model, using a p-value threshold of 10^-4^. Coverage, reads and peaks were visualized with IGV tools.

### Adenoviral infection

For HDAC1 overexpression, overnight cultured islets or INS-1 cells were infected with adenoviruses expressing a full-length rat HDAC1 coding sequence with flag-tagged according to the manufacturer's instructions (GeneChem, Shanghai, China). For HDAC1 knockdown, siRNA targeting the rat HDAC1 gene was constructed using adenovirus expression vector (GeneChem, Shanghai, China). The target sequence used for HDAC1 knockdown was 5'-CTAATGAGCTACCATACAA-3'. Prkaca adenoviruses were constructed with flag-tagged rat Prkaca coding sequence (WT) or lysine 62 mutated to glutamine (K62Q).

### Luciferase reporter assay

After INS-1 cells were plated in 24 wells plates, each well of cells was transfected with luciferase plasmid of rat Tph1 promoter (2067 bp upstream from the transcription start site of Tph1) for 24 h and were then treated with the indicated reagents. pRL-SV40 expressing renilla luciferase (Promega) was used to normalize the luciferase activity. Cells were harvested and luciferase activity was measured using the Dual-Luciferase Reporter Assay System (Promega).

### Statistical analysis

Unless otherwise indicated, all values are expressed as mean ± SEM. Statistical significance was assessed by unpaired Student's t test with a two-tailed distribution for two groups or ANOVA for multiple groups. Differences were considered to be statistically significant when *p*<0.05.

## Supplementary Material

Supplementary figures and tables.
