# Supplementary material for: Protein acetylation derepresses Serotonin Synthesis to potentiate Pancreatic Beta-Cell Function through HDAC1-PKA-Tph1 signaling
Source: Theranostics. 2020 Jun 5;10(16):7351–68. doi: 10.7150/thno.44459 (PMC7330849; doi:10.7150/thno.44459)
Supplement: Supplementary file 1 — Supplementary figures and tables. [file thnov10p7351s1.pdf]

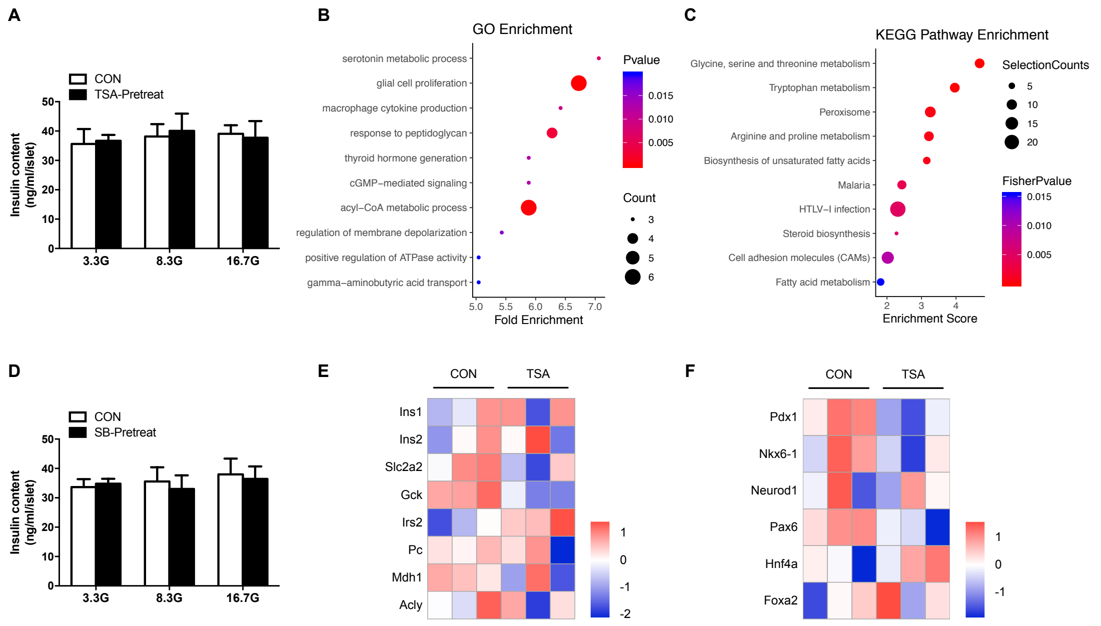

Figure S1. Transcriptome analysis of TSA-treated rat islets. (A) Insulin content of rat islets pretreated with 200 nM TSA for 24 h and stimulated with 3.3, 8.3, and 16.7 mM glucose (3.3G, 8.3G, and 16.7G) for 1 h. (B) GO analysis of upregulated genes by TSA in rat islets. (C) The top 10 most enriched pathways of TSA-upregulated genes in KEGG pathway analysis. (D) Insulin content of rat islets pretreated with 5 mM SB for 24 h and stimulated with 3.3, 8.3, and 16.7 mM glucose for 1 h. (E) Heatmap shows relative expression levels of genes involved in glucose metabolism in TSA-treated islets. (F) Heatmap shows relative expression levels of transcription factors critical for β-cell function in TSA-treated islets.

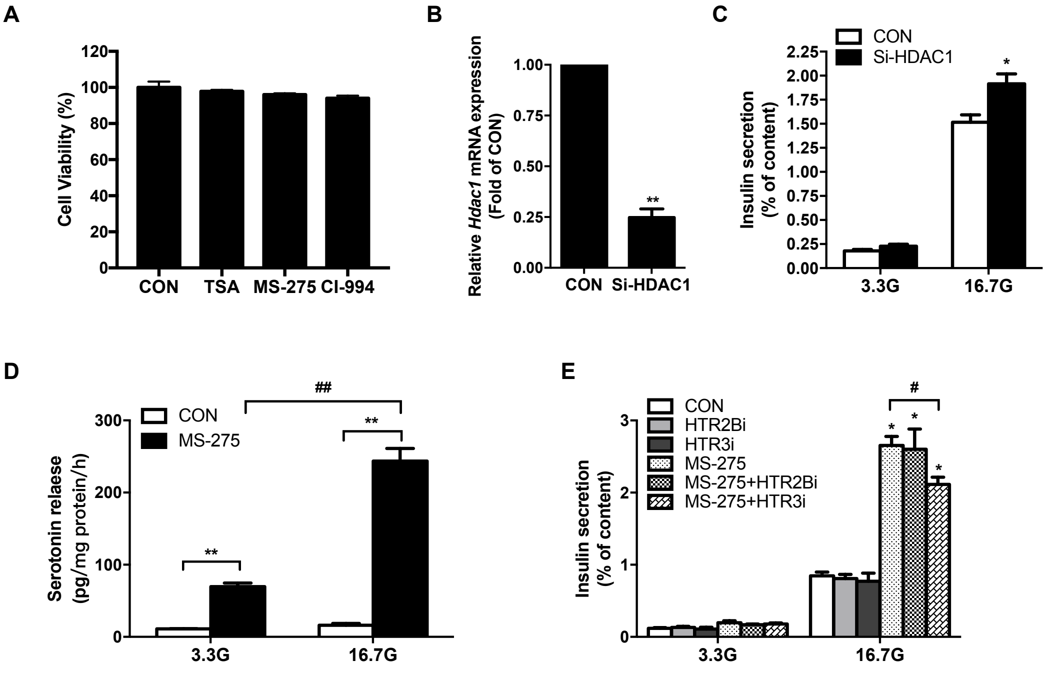

Figure S2. HDAC1 regulates insulin secretion and serotonin release of rat islets. (A) INS-1 cells were treated with 200 nM TSA, 3 μM MS-275, and 10 μM CI-994 for 24 h, and cell viability was determined by CCK-8 assay. (B and C) After rat islets were transfected with control vector (CON) or HDAC1 silencing adenovirus (Si-HDAC1) for 48 h, *Hdac1* mRNA expression and glucose (16.7 mM)-stimulated insulin secretion were detected. (D) Rat islets were pretreated with 3 μM MS-275 at 3.3 mM glucose for 24 h, then stimulated with 3.3 and 16.7 mM glucose (3.3G and 16.7G) for 1 h, and serotonin release was measured. (E) Rat islets were pretreated with or without 3 μM MS-275 in the presence or absence of 1 μM HTR2B inhibitor SB204741 (HTR2Bi) or 100 nM HTR3 inhibitor Ramosetron (HTR3i) for 24 h, and glucose-stimulated insulin secretion was detected. $^{*}P<0.05$, $^{**}P<0.01$ vs. control (CON). $^{\#}P<0.05$ vs. MS-275-16.7G, $^{\#\#}P<0.01$ vs. MS-275-3.3G.

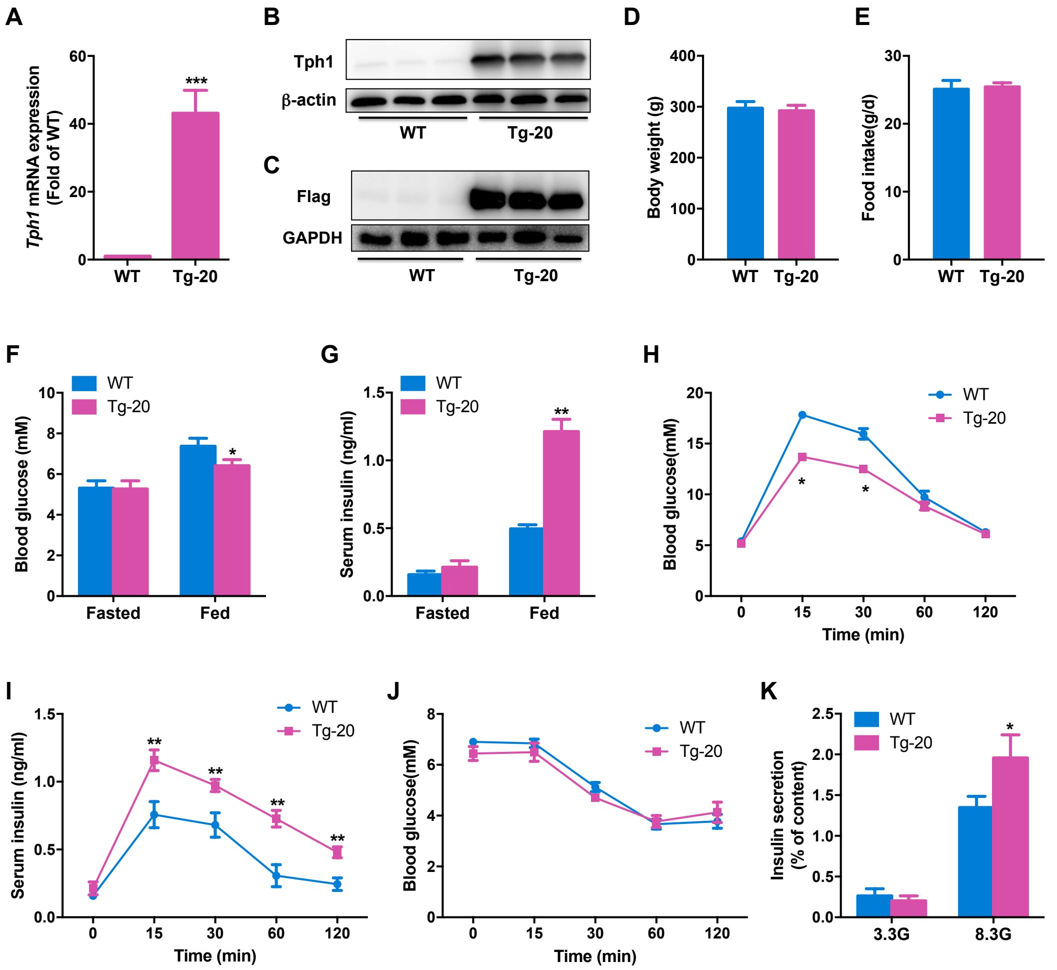

Figure S3. Phenotypes of β-cell-specific Tph1 transgenic rat line #20. (A) Tph1 mRNA levels (*n*=5) in islets isolated from wild-type (WT) and Tph1 transgenic male rat line #20 (Tg-20). (B and C) Tph1 and flag protein levels in islets from WT and Tg-20 rats. Body weight (D) and food intake (E) of WT and Tph1 transgenic rats (*n*=10). (F) Fasted and fed blood glucose levels of WT and Tg-20 rats (*n*=7-10). (G) Fasted and fed serum insulin levels of WT and Tg-20 rats (*n*=7-9). (H and I) Blood glucose (*n*=9-10) and serum insulin (*n*=6) concentrations were measured during IPGTT. (J) Blood glucose levels were measured after insulin injection (*n*=6). (K) Islets isolated from WT and Tg-20 rats were stimulated with 3.3 and 8.3 mM glucose for 1 h, and then insulin secretion was assayed. All the experiments were performed on 10-week-old WT and Tph1 transgenic rats. $^{*}P<0.05$, $^{**}P<0.01$, $^{***}P<0.001$ vs. control mice (WT).

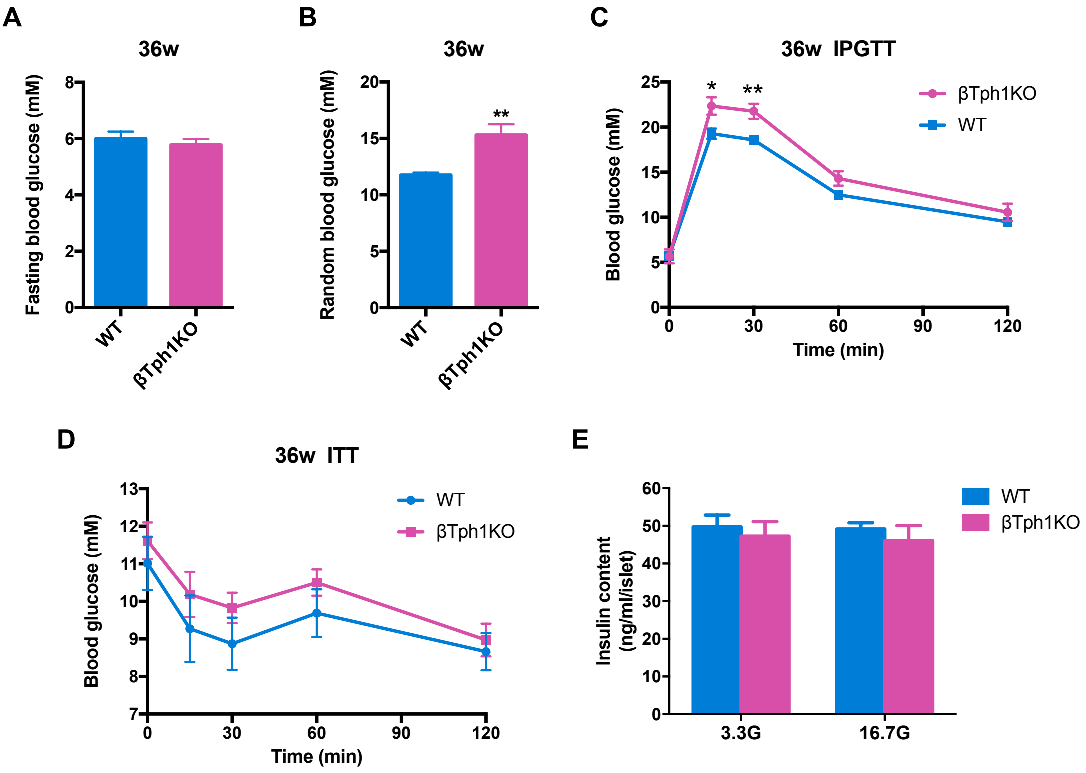

Figure S4. Phenotypes of aging β-cell-specific Tph1 knockout mice. (A and B) Fasting and random blood glucose in 36-week-old WT and βTph1KO mice (n=8-10). (C) IPGTT of 36-week-old WT and βTph1KO mice (n=6). (D) Insulin tolerance test of 36-week-old WT and βTph1KO mice (n=8). (E) Insulin content of islets isolated from 24-week-old WT and βTph1KO mice under 3.3 and 16.7 mM glucose (3.3G and 16. 7G) stimulation (n=3). $^{*}P<0.05$, $^{**}P<0.01$ vs. control mice (WT).

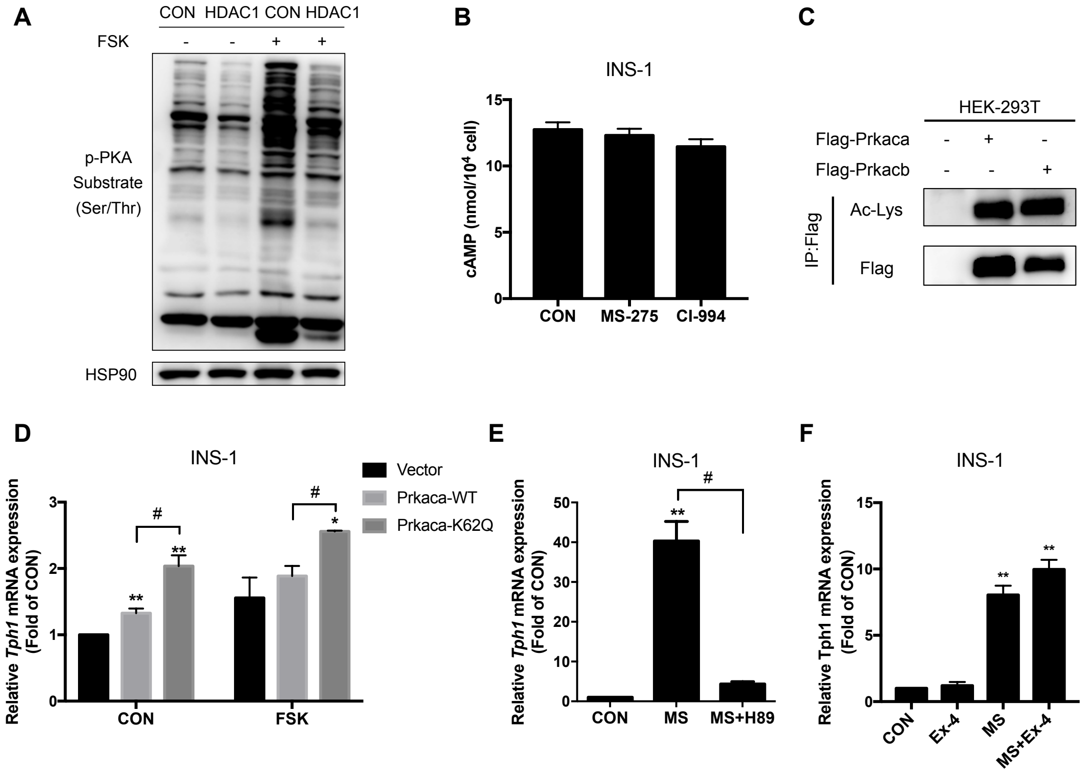

Figure S5. Effect of HDAC1 on β-cell PKA-Tph1 signaling. (A) INS-1 cells were transfected with control vector (CON) or HDAC1-overexpressing adenovirus (HDAC1), and then stimulated with or without 5 μM foskolin (FSK) for 1 h. Phosphorylation of PKA substrate was determined. (B) After INS-1 cells were treated with 3 μM MS-275 and 10 μM CI-994 for 1 h, cellular cAMP levels were determined by ELISA assay. (C) Detection of acetylation levels in ectopically expressed Prkaca and Prkacb in HEK-293T cells. (D) *Tph1* mRNA expression levels in INS-1 cells transfected with control vector, Prkaca wildtype or K62Q adenovirus treated with or without 5 μM forskolin for 24 h. (E) *Tph1* mRNA expression in INS-1 cells incubated with 3 μM MS-275 (MS) in the presence or absence of 10 μM H89 for 24 h. (F) *Tph1* mRNA expression levels in INS-1 cells incubated with 10 nM Exendin-4 and 3 μM MS-275 for 24 h. Data are expressed as mean ± SEM of three independent experiments. $^{*}P$<0.05, $^{**}P$<0.01 vs. Vector or control (CON). $^{\#}P$<0.05.

Table S1. Sequences of primers for RT-PCR

Gene name	Species	Forward	Reverse
Tph1	Rat	TGCGACATCAACCGAGAA	GCAGAAGTCCAGGTCAGAAAT
Tph2	Rat	CAGGGTTACTTTCCTCCATCG	AGCAGGTTGTCTTCGGGTCA
Ddc	Rat	CCCAGGAGCCAGAAACATA	GGGGAAGTAAGCGAAGAAGT
18S	Rat	CACGGGTGACGGGGAATCAG	CGGGTCGGGAGTGGGTAATTTG
Tph1	Mouse	GAAGACAACATCCCGCAACT	AAAGGCTAACCCCGACAGA
Actin	Mouse	TGTACCCAGGCATTGCTGAC	CTGCTGGAAGGTGGACAGTG
